# Biocomposite Active Whey Protein Films with Thyme Reinforced by Electrospun Polylactic Acid Fiber Mat

**DOI:** 10.3390/foods14010119

**Published:** 2025-01-03

**Authors:** Andreea (Lanciu) Dorofte, Iulia Bleoanca, Florentina Ionela Bucur, Gabriel Mustatea, Daniela Borda, Felicia Stan, Catalin Fetecau

**Affiliations:** 1 Bioaliment TehnIA Food Research Center, Faculty of Food Science and Engineering, Dunarea de Jos University of Galați, Domnească Street, No. 111, 800201 Galați, Romania; andreea.dorofte@ugal.ro (A.D.); iulia.bleoanca@ugal.ro (I.B.); florentina.bucur@ugal.ro (F.I.B.); 2 National Research and Development Institute for Food Bioresources—IBA, Ancuta Baneasa No 5. Street, Sector 2, 020323 Bucharest, Romania; gabi.mustatea@bioresurse.ro; 3 Center of Excellence Polymer Processing, Faculty of Engineering, Dunarea de Jos University of Galați, Domnească Street, No. 111, 800201 Galați, Romania; felicia.stan@ugal.ro (F.S.); catalin.fetecau@ugal.ro (C.F.)

**Keywords:** active films, essential oils, fibers, electrospinning, whey protein, controlled permeability

## Abstract

Electrospinning is a versatile technique for obtaining nano/micro fibers which are able to significantly change the active properties of composite materials and bring in new dimensions to agri-food applications. Composite bio-based packaging materials obtained from whey proteins, functionalized with thyme essential oil (TEO) and reinforced by electrospun polylactic acid (PLA) fibers, represent a promising solution for developing new active food packaging using environmentally friendly materials. The aim of this study is to obtain and characterize one-side-active composite films covered with a PLA fiber mat: (i) WF/G1, WF/G2, and WF/G3 resulting from electrospinning with one needle at different electrospinning times of 90, 150, and 210 min, respectively, and (ii) WF/G4 obtained with two face-to-face needles after 210 min of electrospinning. While TEO bioactivity is mainly related to its antimicrobial and antioxidant properties, the PLA fiber mat uplifted the composite mechanical and barrier properties of films. The bi-layer films obtained were characterized by SEM, showing the distribution of the electrospun fiber mat and an increased thickness of the PLA layer from WF/G1 to WF/G4, while FTIR spectra showed the structural vibrations of the functional groups. The experimental results show that WF/G4 have a FTIR fingerprint resembling PLA, retained ~50% of the volatile compounds present in the uncovered film (WF/TEO), while it only had 1.41 ± 0.14 (%) of the permeability to octanol of the WF/G1 film. WF/G4 exhibited 33.73% of the WVP of WF/G1 and displayed the highest tensile strength, about 2.70 times higher than WF/TEO. All films studied revealed similar antimicrobial effect against *Bacillus cereus*, *Geotrichum candidum*, and *Rhodotorula glutinis* and good antiradical activity, thus demonstrating good prospects to be applied as food packaging materials. WF/G composite materials are good candidates to be used as bioactive flavoring primary packaging in hard cheese making.

## 1. Introduction

Lately, the interest in alternative packaging solutions to current petroleum-derived polymer ones used by the food industry furthered the quest for new bio-based packaging solutions, driven by the need for sustainable, more effective, healthier, and safer food production systems than the current ones. The main advantages of the bio-based polymers refer to their sources, which are either renewable, low-cost, or are underused byproducts with high biodegradability, possessing good capacity to be functionalized (e.g., with antimicrobial or antioxidant compounds) and prospects to be used as edible, active, or modified atmosphere packaging. Bio-based packaging materials obtained from natural raw materials are classified as class I (proteins, polysaccharides, or lipids), class II (i.e., polylactic acid (PLA), bacterial cellulose), and class III (produced by fermentation).

Despite the multiple advantages of class I bio-based polymers, which are considered to be competitive eco-friendly food packaging alternatives, there are also several drawbacks, mainly related to their low mechanical strength, higher water vapor permeability, and poorer thermal properties compared to conventional plastic packaging, that limit their commercial use in food applications. Thus, composite bio-based polymers resulting from combining different types of biopolymers could provide key characteristics to packaging materials, uplifting their poor mechanical, physical, and thermal properties.

Whey proteins (WPs) are used for the development of biodegradable packaging materials due to their considerable large availability, low cost, good biodegradability, flexibility, neutral taste and flavor, and medium moisture permeability. Nonetheless, the barrier properties against gases and aroma are considered rather poor, while mechanical properties are low in comparison to conventional polymers [1,2].

Numerous studies investigated the use of antimicrobial essential oils to enhance the technological functional properties of whey protein polymers. For instance, blends of cinnamon and rosemary [3] have been shown to increase the antioxidant properties of films. Additionally, oregano, rosemary, and garlic essential oils [4], as well as lemon and bergamot essential oils [5], have been utilized to provide biopolymers with antimicrobial properties. Other studies have explored combinations of EOs and antimicrobial peptides (i.e., oregano oil, garlic oil, nisin, and natamycin) applied as layers on sliced Kasar cheese [6]. Furthermore, essential oils such as lemongrass and lemon are used in order to improve the product storage time of fresh-cut pears [7] or zucchini [8].

The reinforcement of biopolymers with natural fibers, achieved by layering using fibers into the polymer matrices, determine an increase in strength and stiffness of the biopolymer structure, resulting in green hybrid composites. Researchers working on natural fiber reinforcement with PLA composites have reported significant benefits regarding recyclability, renewability, durability, processability, and compostability [9].

Polylactide or PLA is a polymer produced from lactic acid by polymerization, typically resulting from fermented starchy plants like corn, potatoes, sugarcane, cassava, or sugar beet pulp. PLA, a recyclable and renewable hydrophobic aliphatic polyester, shows high rigidity and excellent processability [10]. Electrospinning was used to transform PLA solution into fine fibers under a high-voltage electric field. This technique produces fibers with diameters ranging from micro to nanometers by stretching a solution of polymer in a high electric field. PLA demonstrated ease in fiber production by electrospinning and displayed high transparency and high biotic and abiotic degradability for 45–60 days at 50–60 °C [11]. Several applications of electrospun fibers have been developed as carriers of active substances, environmental bioremediation solutions, or tissue engineering, but their use in bio-based food packaging materials is also in the crosshairs [12].

However, electrospinning has not yet reached the maximum level of readiness for industrial applications, as requested by the market, due to scalability challenges [13,14].

The fiber mat resulting from crosslinking the PLA fibers in an electrospun mat is expected to enhance the composites’ mechanical strength, providing better barrier properties and optical clarity [14]. Recently, new active composite materials with a PLA fiber mat were researched [15,16,17,18,19].

The main advantage of using an electrospun fiber mat, besides its cost-effectiveness and the large-surface-area-to-mass ratio [14,20], lies in its potentially dynamic role of EOs’ controlled permeation through the fiber mat. Therefore, if controlled permeation through composite materials could be achieved, it would provide flavor and protection to foods on the active side of the packaging, ensuring the retention of the main active terpenes, followed by their diffusion into the food matrix. On the other side of the packaging, the limited Eos’ permeability would prevent contamination, ensuring long food storage stability. However, to the best knowledge of the authors, aspects regarding the controlled permeability of EOs incorporated into composite packaging materials with an electrospun fiber mat have not been studied up until now.

The objective of this work was to obtain biocomposite bi-layer hybrid films with whey proteins (WF) and thyme essential oil (TEO) covered by a PLA fiber mat (WF/G), and to assess the mechanical properties and their capacity to significantly reduce the water vapor, oxygen, and carbon dioxide permeability in comparison with WF. Moreover, the controlled permeation of the volatile embedded TEOs in the WF through the PLA fiber mat as a function of the mat thickness and its influence on antimicrobial and antiradical properties was evaluated.

## 2. Materials and Methods

### 2.1. Materials

The composites obtained in this study are based on whey protein concentrate ProMilk 802FB, kindly provided by KUK-Romania (Bucharest, Romania). A semicrystalline PLA polymer obtained from renewable resources, with a d-lactide content of 4% (96% of l-lactide), 66.000 g/mol, was purchased from IMAKE3D, Smart Materials 3D (Bucharest, Romania). TEO (*Thymus vulgaris*) was purchased from Aroma Zone (Provence, France); helium (99.996% purity) was obtained from Linde Gaz S.R.L., (Galati, Romania); 2, 2-diphenyl-2-picrylhydrazyl (DPPH), chloroform (99.5%), and 2-octanol (97%) were purchased from Sigma-Aldrich (Steinheim, Germany); acetone was obtained from Cristal R Chim (Bucharest, Romania); anhydrous glycerol (98% purity) was purchased from Redox SRL (Bucharest, Romania); and test microorganisms (*Bacillus cereus* ATCC 10876, *Geotrichum candidum* MIUG M166, and *Rhodotorula glutinis* MIUG D164) were obtained from the MIUG collection, Dunărea de Jos University of Galaţi, Romania. *Listeria monocytogenes* Scott A (CIP 103575, serotype 4b) was provided by the Center of Biological Resources, Pasteur Institute, Paris, France.

### 2.2. WF/TEO Film Preparation

Whey protein films (WF) were obtained by casting following the protocol published in [2] with slight modification. The film-forming solutions were functionalized with TEO (3% *w*/*w*). This plant essential oil was chosen due to its high carvacrol content, thymol, and p-cymene, and other well-known terpenes with stronger antimicrobial properties in comparison with other EOs (i.e., cinnamon, bay leaf, lavender, clove), as demonstrated by a screening study performed by our group. The effective TEO concentration was based on results from other similar previous studies [2,8,21,22]. The sonication treatment, degassing process, and the drying methods applied were similar to the protocol previously described in [2]. The films were preconditioned at 25 ± 1 °C and 50% RH using propylene glycol placed in glass desiccators for 24 h.

### 2.3. Electrospinning of PLA Fibers

PLA pellets (10% *w*/*w*) were accurately weighed and solubilized in 3:1 chloroform- acetone mixture, under magnetic stirring, at 50 °C and 250 rpm, until a transparent viscous solution was obtained. The electrospinning process was carried out using a fiber electrospinning unit (TL-Pro-BM, Tong Li Tech, Shenzhen, China) at 22 ± 1 °C and 30 ± 5% RH. The solution was loaded in a 25 mL syringe with 19-gauge steel needle connected to the positively charged electrode. Pre-prepared whey films were previously mounted onto a collector and the PLA solution was directly fed in the form of a fibrous layer at a flow rate of 0.5 mL/h. A horizontal in-motion slider for spinneret/spinnerets with a scanning speed of 20 mm/s was used. Other electrospinning parameters included a collector rotation speed of 20 rpm and the voltage applied of (+) 15 kV, (−) 8 kV. The electrospinning parameters and system configuration were selected based on preliminary experiments performed prior to this study.

**Figure 1:**
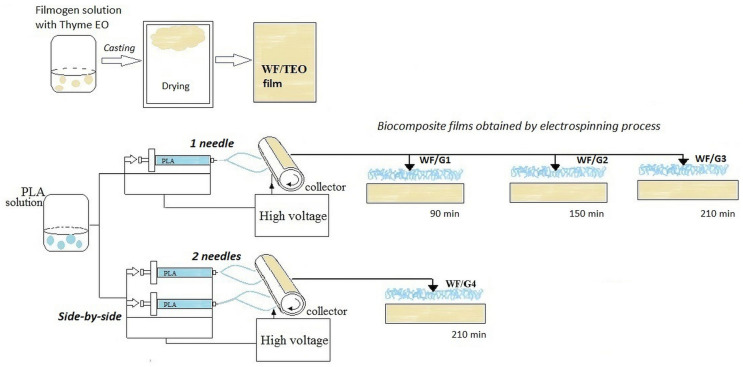
Electrospinning process.

Finally, four films were obtained at different electrospinning times, with the following configurations: (i) one spinneret placed at a 30 cm distance between the needle tip and the collector and an angle between the spinneret and drum collector generator of 75° for producing WF/G1 (90 min, 1 pump on), WF/G2 (150 min, 1 pump active), and WF/G3 (210 min, 1 pump active) films, and (ii) a dual set up with independent channels, two syringe pumps, and two face-to-face spinnerets directed towards each side of the collector and with the spinneret tilted at 75° on both sides of the drum collector generator, each at 30 cm from the collector, in the case of WF/G4 (210 min, 2 pumps active) (Figure 1).

### 2.4. Characterization of Electrospun Bi-Layer Films

#### 2.4.1. Morphological Characterization

The morphological characterization of the bi-layer WF film with a PLA fiber mat was carried out using Scanning Electron Microscopy (SEM) (Quanta 200, Thermo Fisher Scientific, Waltham, MA, USA). The films, vacuum-coated with a thin layer of gold, were analyzed at 19 KV voltage and magnification 2000× for surface and for cross-section images at 15 kV and 1000× for all WF/G films. ImageJ version 1.49v, Java 1.8.0_45 (NIH, Bethesda, MD, USA) analysis software was used for the quantification of 150 minimum Feret fiber diameters and mat thickness in at least three different film areas.

#### 2.4.2. FT-IR Spectrometry

The method applied was described in detail [2,23]. The infrared spectra of the WFs were analyzed using a Nicolet iS50 FT-IR spectrometer (Thermo Scientific, Waltham, MA, USA) assembled with ATR, KBr beam splitter, and DTGS detector. A number of 32 scans in the 4000–400 cm^−1^ range with 4 cm^−1^ resolution were performed using air as background. Calibration of ATR-FTIR equipment was performed with 2-propanol as internal standards for hydroxyls functional groups with a detection limit of 0.5% (*w*/*w*).

#### 2.4.3. Determination of Moisture Content and Solubility in Water

Moisture content (***MC***) was measured gravimetrically, as proposed by [2]. Briefly, the film samples were weighted (***W*_0_**) into glass crucibles, dried in an oven at 110 °C for 24 h, and, after cooling at room temperature, were weighted again (***W_i_***). Moisture content for each film was determined in triplicate by means of Equation (1).

(1) MC=W0−WiW0×100 (%)

Solubility in water (***S***) was determined by the method described by [2]. Three weighted samples of each film were immersed in beakers containing 30 mL of distilled water at 25 °C for 24 h with occasional stirring. After removal from water, the films were patted with filter paper, further dried at 110 °C to a constant weight (***W_f_***), then cooled and re-weighed. To determine the solubilized dry matter, Equation (2) was applied:

***S*** = [(***W_i_*** − ***W_f_***)/***W_i_***] × **100** (%) (2)

where ***W_i_*** is the initial dry weight and ***W_f_*** is the final dry weight.

### 2.5. Barrier Properties of Biocomposite Films 

#### 2.5.1. Volatile Fingerprint

The volatile fingerprint was analyzed using a gas chromatograph (Trace GC Ultra) (Thermo Scientific, Waltham, MA, USA) coupled with a mass spectrometer with ion trap MS ITQ 900 (Thermo Scientific, Waltham, MA, USA). An SPME system was used to extract the gaseous phase from samples with a DVB/CAR/PDMS adsorption fiber. The method of incubation, extraction, and analysis was previously optimized and described in detail [22].

**Figure 2:**
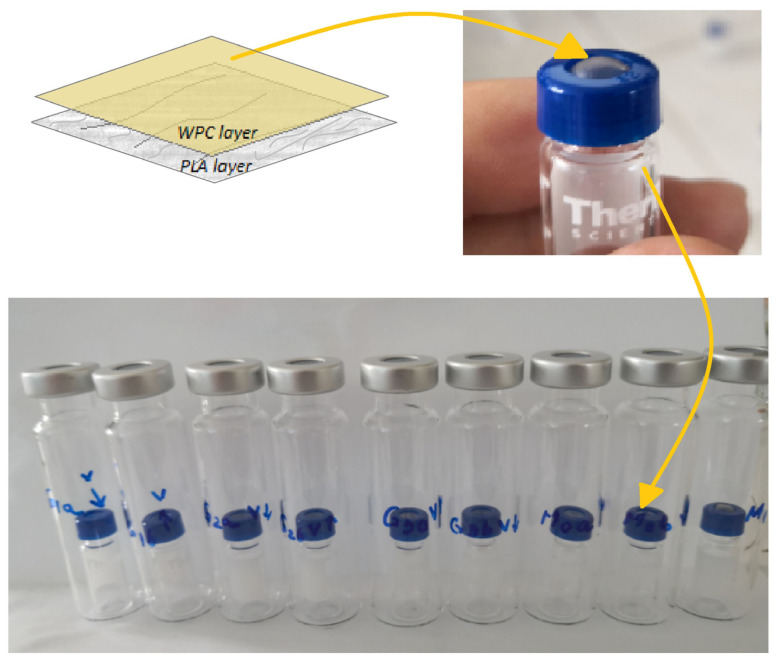
Separation of VOCs through the WF/Gs with PLA fiber mat applied during SPME GC/Ms analysis using two glass vials.

In order to establish the retention capacity of volatile compounds from TEO by the composite consisting of WF covered with PLA fiber mats of different thickness, the tested biocomposite films were mounted on a small vial lid (Figure 2) with the PLA mat oriented upwards and the WF film with TEO oriented downwards. Thus, a more clear-cut separation surface between the two vials was created, a small 2 mL glass vial and a larger 20 mL one. The WF samples were spiked with 5 μL of 2-octanol (0.651 mg/mL) used as the internal standard.

Further on, the small vials were closed with a lid formed by the films with a WF, TEO, and PLA mat on the top and, later on, were inserted into the larger 20 mL vials, which were closed and incubated at 50 °C for 20 min (Figure 2). The SPME fiber was inserted into the large vial, above the small glass vial, and exposed only to the volatile compounds present in the large glass container, which were further adsorbed by the fiber for 20 min.

The adsorption–desorption procedure from the fiber and the temperature ramp selected is similar to the one described by other researchers [23].

The tentative identification of volatile compounds (VOCs) was performed using NIST 08 library database provided by the Xcalibur^TM^ 2.1 (Thermo Fisher Scientific, Waltham, MA, USA) software, and the relative VOC concentration expressed semi-quantitatively was estimated using the method applied by researchers [2,8,21] considering the volume of WF/G films through which VOCs migrated (μg octanol/cm^3^).

#### 2.5.2. Permeability to Octanol

Using the same experimental set-up as described in Section 2.5.1, a supplementary amount of 5 μL 0.651 mg/mL of 2-octanol was introduced into the small vial to assess the permeability of the PLA fiber mat from the WF/G films in comparison with the WF/TEO films.

#### 2.5.3. Water Vapor Permeability of EFs

Water vapor permeability (***WVP***) was determined following a procedure described in detail [23] using Equation (3):

(3) WVP=slope×xA×Δp  (g×mm/mm2×h×Pa)

where slope is the weight loss of the tube /time, (g/s); ***x*** is the average thickness of the film, (mm); ***A*** is the area of the exposed film, (m^2^); and **∆*p*** is the vapor pressure difference across the film, (Pa).

#### 2.5.4. Gas Permeability of EFs

The gas barrier properties of the films were tested using the manometric method with Dansensor Lyssy L100-5000 (Ringsted, Denmark) equipment with a cooling system with thermostat for two different dry gases (i.e., O_2_ and CO_2_). The characteristics of the process were a power supply of 230 VAC, 50 Hz, a measuring scale calibrated from 1 to 10,000 mL/m^2^/24 h, two independent measuring chambers, a two-step rotary vacuum pump, and a built-in thermal printer. A film sample of 80 mm in diameter was placed on a stainless-steel mask. The test sample was attached to the self-adhesive sample card, which was then positioned within one of the test chambers, creating a separation between the upper and lower chamber. The operating conditions were set at 23 °C and 0% relative humidity.

The sample’s measured time interval was compared with the calibration time of a standard film with known permeability, resulting in the gas transmission rate being expressed in cm^3^/m^2^·24 h·bar. Test cycles were repeated until maximum repeatability of the results was obtained, indicating that the equilibrium of the samples was reached.

### 2.6. Color and Opacity Analysis

The color of the film samples was determined using a Minolta colorimeter (Model CR410, Tokyo, Japan). The films placed on a white surface were used to determine the CIELab color space parameters as L^*^ (luminosity), a^*^ (green to red), and b^*^ (blue to yellow) parameters [8]. Total color change **Δ*E*** was determined using Equation (4). Values of Chroma (***C_ab_***) and Hue angle (***H_ab_***) were calculated according to Equations (5) and (6), respectively, using values representing the mean of three replicates:

(4) $$\Delta E=\sqrt{{\left(L^{*}-L_{c}\right)}^{2}+{\left(a^{*}-a_{c}\right)}^{2}+{\left(b^{*}-b_{c}\right)}^{2}}$$

(5) $$C_{ab}=\sqrt{{a^{*}}^{2}+{b^{*}}^{2}}$$

(6) Hab=arctanb*a* (°)

where L^*^, a^*^, b^*^ are the color parameter values of the sample and ***L_c_***, ***a_c_***, ***b_c_*** are the corresponding values for control WF without fibers.

Opacity (O) was determined by colorimetry with Equation (7):

(7) O=LbLw×100 (%)

where ***L_b_*** is the luminosity of the WF measured with a black background and ***L_w_*** is the luminosity of the WF measured with a white background.

### 2.7. Mechanical Characterization

Each film was cut into 1 × 10 cm rectangular specimens and then subjected to tensile testing at a crosshead displacement of 50 mm/min. The mechanical properties of the equilibrated films (elastic modulus or stiffness and maximum tensile strength) were determined at 22 ± 0.2 °C and 50 ± 0.1% RH. Tests were carried out with a testing machine with 0.025 N accuracy and a displacement reading resolution of 0.001 mm (M350-5AT Testometric, Rochdale, UK) [24].

It should be noted that the thickness of the film was determined as the average of 10 measurements with an electronic caliper (BurgWachter, Wetter, Ruhr, Germany) within a measurement range of 0–150 mm and a precision of 0.02 mm.

### 2.8. Functional Properties of Whey Films with TEO (WF/TEO)

#### 2.8.1. Antimicrobial Activity of WFs

The antimicrobial activity of WFs containing TEO was analyzed by means of the disk diffusion method [22] against four microorganisms, *Bacillus cereus* ATCC 10876, *Geotrichum candidum* MIUG M166, *Rhodotorula glutinis* MIUG D164, and *Listeria monocytogenes* Scott A. The microorganisms were selected considering the whey protein structure of the composite materials and potential applications in the dairy industry where thermoresistant spore-forming bacteria such as *B. cereus* might be present, and often fungi like *G. candidum* and *R. glutining* thrive, causing quality losses. Furthermore, the pathogen *L. monocytogenes* raises many safety concerns in the dairy industry.

Brain heart infusion agar (1.5% agar) was used for assessing listericidal effect, plate count agar for bactericidal activity, and rose bengal chloramphenicol agar (Oxoid Ltd., Basingstoke Hampshire, UK) for fungicidal evaluation. Previously, MIC and MBC of TEO were determined and reported [23]. Inoculum of each of the four tested microorganisms was prepared by overnight culturing at 27 °C for fungi or 37 °C for bacteria. The four test microorganisms were then inoculated onto specific culture media with approx. 10^6^ CFU/mL, over which films of 1 cm diameter were placed. Following incubation, the inhibition diameters surrounding the film disks were estimated with a digital caliper (Burg Wachter, Germany). Three independent experiments were performed and the average inhibition diameter of the inhibition zones ± SD were reported.

#### 2.8.2. DPPH Radical Scavenging Activity

The antiradical capacity of WFs was estimated using the DPPH assay. Briefly, 0.1 g of film sample was placed in 10 mL of ultrapure water and sonicated (3 min) to favor TEO extraction in aqueous phase. Then, the mixture was kept for 12 h at 22 °C. Subsequently, the supernatant was collected and 2 mL of the supernatant was mixed with 4 mL of DPPH solution (0.1 mM) [25] and stored in the dark. After 30 min, the absorbance at λ_max_ = 517 nm was measured with a UV–Vis spectrophotometer (UV VIS Cintra 202, Braeside, Australia). The results are expressed as% of inhibition (I) of radical scavenged activity (AA) using Equation (8):

(8) I=Ac−AsAS×100 %

where ***A_C_*** and ***A_S_*** are the absorbance of free DPPH solution and the absorbance of DPPH solution with WFs, respectively. Linearity of Trolox calibration curve was established in the range 10 to 1000 μM (TE/g).

### 2.9. Statistical Analysis

The data represent the mean of three independent replicates ± standard deviation (SD). The independent factorial analysis of variance was performed by one-way ANOVA and Tukey’s post hoc test for establishing potential significant differences among groups at *p* values < 0.05.

The FT-IR spectra were pre-processed using multiplicative scatter corrections (MSC) and overlapped. Principal component analysis (PCA) with Varimax rotation was performed using Unscrambler software (Version 9.7, CAMO, Oslo, Norway) to evaluate the main factors’ contribution in explaining the total variance. The cut-off criterion considered the reduction in the total variance.

## 3. Results

### 3.1. Characterization of Electrospun Bi-Layer Films 

#### 3.1.1. Visual and Morphological Analysis

**Figure 3:**
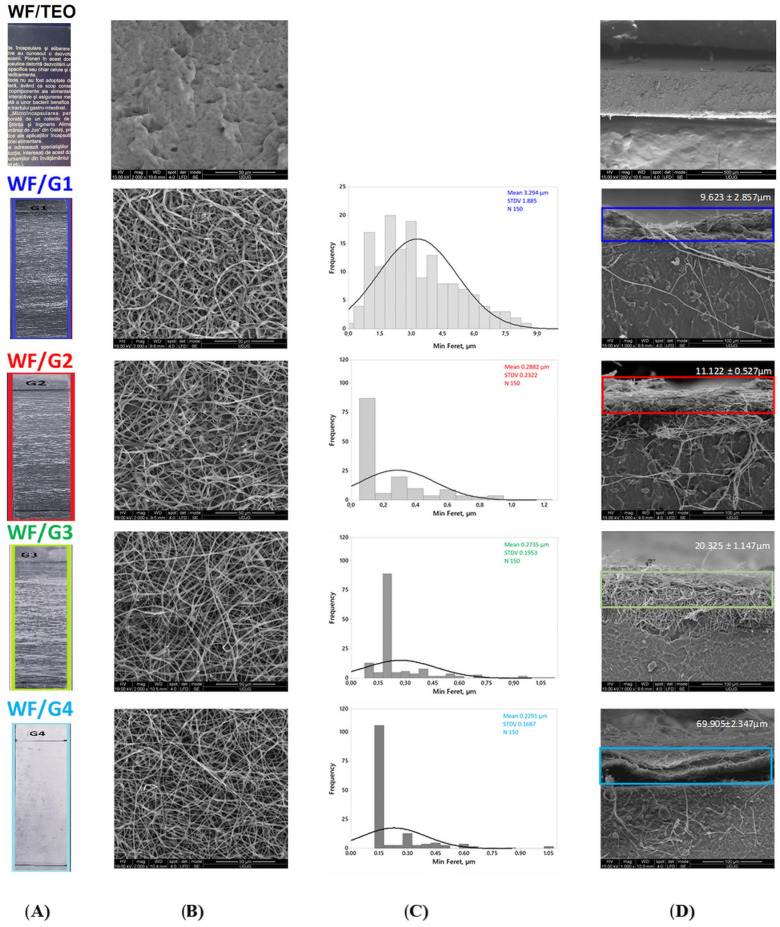
Visual and morphological analysis of WF/G1–G4 fiber layers: (**A**) macroscopic view (1:2); (**B**) SEM top view of PLA fiber mats; (**C**) histograms of minimum Feret fiber distribution evaluated from N = 150 measurements; (**D**) SEM cross-section with the estimated thickness of the mat given as mean ± standard deviation (SD).

Electrospinning produces fibers with the diameter depending on several factors such as polymer type, polymer chain conformational structure, viscosity, polarity, electrical conductivity, and surface tension [26]. In this study, all PLA fibers had a homogeneous structure visible with the naked eye (Figure 3A) and without agglomerations in spheroid beads (Figure 3B), indicating that adequate electrospinning conditions were reached. SEM evaluation of the biocomposite films’ surface revealed that the PLA fibers have a cylindrical shape with a smooth surface. Their minimum Feret diameter presented in Figure 3C ranges from 3.294 ± 1.885 μm for WF/G1 to 0.229 ± 0.169 μm for WFG4, but random nanofibers with a smaller diameter, around 800 nm, are also present (Figure 3C).

The differences in average fiber diameter with increased electrospinning time could be justified by the more uniform fiber distribution and increased density in the mat structure, as demonstrated by the decrease in the SD from WF/G1 to WF/G4. Basically, longer electrospinning times produced denser and more uniform fiber mats (Figure 3A). Nonetheless, limitations associated with measurements being completed manually using ImageJ 1.49v, Java 1.8.0_45 software should also be considered [27].

Researchers [28] also reported a DOE model showing a negative correlation between composite PLA-PCL fiber diameter as the dependent variable and time as the independent variable, considering only 1 min and 10 min of electrospinning, whereas the increase in thickness was correlated with an increase in elongation and tensile strength. In this case, the SEM analysis indicates the presence of the interwoven nanofibrous layers (Figure 3B). The cross-view of the films shows different thicknesses of the PLA fibrous layer ranging from 9.623 ± 2.855 μm (WF/G1) to a thickness approx. seven times higher, of 69.905 ± 2.347 μm (WF/G4), while WF/G3 is two times higher than WF/G1. The WF/G4 fiber mat, delivered by two needles with two pumps compared to the other WF/Gs, is maintained as two independent layers (Figure 3D).

A relevant characteristic of composites covered by a PLA fiber mat is the ability to completely biodegrade components with non-ecological toxicity risk. The biodegradation of electrospun PLA-based materials, by in-soil degradation, industrial composting conditions under highly controlled conditions of temperature and humidity, or by anaerobic digestion, has been recently reviewed [29].

#### 3.1.2. FT-IR Analysis

**Figure 4:**
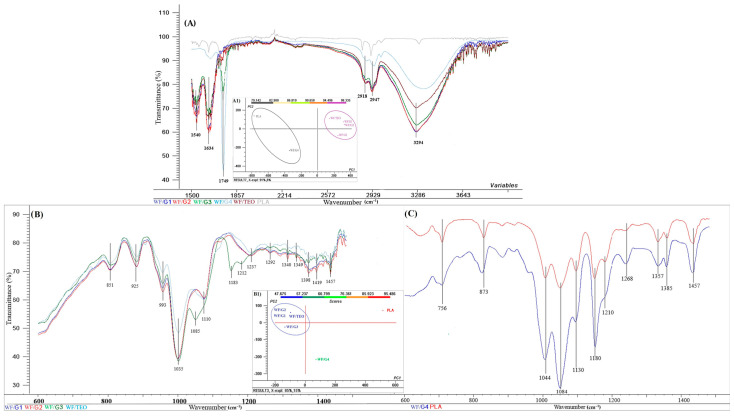
Aligned FT-IR spectra of (**A**) WF/G1, WF/G2, WF/G4, WF/TEO, and PLA from 1500 to 3400 cm^−1^ wavenumber and PCA scores and clusters; (**B**) WF/G1, WF/G2, WF/G3, and WF/TEO and (**C**) WF/G4 and PLA from 600 to 1500 cm^−1^ wavenumber; (**A1**) the principal component analysis (PCA) scores and clusters of WF/G1, WF/G2, WF/G4, WF/TEO, and PLA spectra for 1500 to 3400 cm^−1^; and (**B1**) the PCA scores and clusters of WF/G1, WF/G2, WF/G4, WF/TEO, and PLA spectra in the fingerprint region (600 to 1500 cm^−1^).

For a better analysis of the films, the spectra were divided into two intervals: analytical region above 1500 cm^−1^ (Figure 4A) and fingerprint region from 600 to 1500 cm^−1^ wavenumber (Figure 4B,C). For the region above 1500 cm^−1^ wavenumber, as can be seen from the FT-IR analysis in Figure 4A, the WF/G4 film (light blue) has a different characteristic structure compared to the other biocomposite films, namely WF/G1, WF/G2, and WF/G3. The alure of the WF/G4 spectrum is more closely related to the PLA spectrum than to G1, G2, and G3, which are closer to the WF/TEO spectrum This can also be noticed from the PCA analysis with two principal components that explain 99% of the total variance (Figure 4A1). However, the principal component PC1 explains most of the system variation (91%) on abscissa, while PC2, placed on the ordinate, explains only 8% of the total variation.

Two clusters resulted from the score plot, one in which WF/G4 and PLA are grouped and one with WF/G1, WF/G2, WF/G3, and WF/TEO are gathered in the same quadrant (Figure 4A1). Thus, it can be inferred that, once the thickness of the PLA fiber mat is higher, the spectra are less influenced by the whey proteins and TEO functional groups present in the structure of composites and more influenced by the PLA properties. The PLA fiber mat seems to hinder some specific vibration present in WF/TEO and WF/G1, WF/G2, and WF/G3 films. However, it should be considered that penetration depth has the same magnitude order as the infrared light wavelength, and this makes ATR-FTIR less sensitive to some bioactive compounds [30].

Moreover, it is noticeable that WF/G1 and WF/G2 spectra overlap for the entire interval. The broad peak between 3280 and 3370 cm^−1^ is associated with the vibration of the hydroxyl groups [14] present in the WF/G1, WF/G2, and WF/G3, and in WF/TEO with a maximum at (3924 cm^−1^), and it is very weak in PLA and has a blue shift at 3298 cm^−1^. It can also be noticed that, when the thickness layer of PLA fibers increases, particularly in WF/G3 and, more obviously, in WF/G4, the intensity of OH vibrations are less visible in the spectra. Then, two peaks in the range of 2850–2930 cm^−1^, corresponding to aliphatic C–H stretching noticed in this study, were also reported by other researchers studying PLA-Thyme and guar gum nanofibers [31] and in PLA-cinnamon microcapsules reported by [32]. In this research, control film (PLA) and WF/G4, dominated mostly by the PLA vibrations, exhibited a low intensity peak at 2947 cm^−1^ associated with C-H stretching, while, at the same wavenumber, the peak had higher intensity in composite films WF/G1, WF/G2, WF/G3, and WF/TEO, indicating a higher exposure of aliphatic chains due to complex interactions between proteins, essential oils, and PLA mat. The thicker the PLA mat, the lower the intensity peak registered at 2947 cm^−1^, supposedly because a higher number of hydrogen bonds were formed between the phenolic hydroxyl groups in TEO and the hydroxyl groups in PLA.

The increased peak intensity at 1634 cm^−1^ of composites WF/TEO, WF/G1, WG/G2, and WF/G3 shows that a higher proportion of carbonyl groups were present in the composites mainly due to the incorporation of essential oil in WF films, in comparison with the PLA sample, while in the WF/G4 sample with the thickness PLA mat, the vibrations were less intense. The amide I spectral region (1600–1700 cm^−1^) is the most sensitive region to the structural components of the proteins [33]. This region also indicates the formation of higher number of hydrogen bonds via an intensity decrease from 1650 to 1630 cm^−1^ and shift to lower frequencies, as observed in WF/G3 and WF/G2 in comparison with the WF/G1 sample. Presumably, more hydrogen bonds were formed between the essential oil, the protein matrix, and the PLA mat in WF/G3 and WF/G2 in comparison with the WF/G1 sample and this observation correlates with the films’ mechanical strength. WF/G4 and the PLA samples displayed similar behavior in this wavelength range, different from the rest.

All the analyzed samples displayed spiked peaks at 1749 cm^−1^ associated with the stretching of the carbonyl group, with a higher intensity in the PLA sample but also in WF/G4 and WF/G3, with WF/G4 having the thickest PLA mat among all samples.

The vibrations with spikes at 1540 cm^−1^ were related to bending groups of N–H (amide type II) visible for WF/G1, WF/G2, WF/G3, and WF/TEO spectra and less evident in WF/G4 and PLA, while at 1634 cm^−1^, the vibrations were associated with the unsaturation of the benzene ring, also identified in other studies [32].

The IR fingerprints of the films with only a thin mat of PLA fiber (WF/G1, WF/G2, WF/G3) and the control (WF/TEO) are represented on the left of Figure 4B, while on the right, WF/G4 and PLA spectra are represented Figure 4C. At 756 cm^−1^ and 870 cm^−1^, the vibrations in the PLA units of polymer, visible on the right of the graph (Figure 4C), were also previously reported [32].

The smooth and rather weak peaks at 851 cm^−1^ associated with =CH_2_ bending (wagging) are similar in WF/G1, WF/G2, WF/G3, and WF/TEO (Figure 4B). At 951 and 993 cm^−1^, the peaks are showing the bending of -HC=CH- isomers cis and trans, respectively [34,35]. The peak at 1083 cm^−1^, present in WF/G3, is rather specific for the PLA as results from the graph (Figure 4C) placed on the right (1085 cm^−1^). Peaks at 1035 and 1110 cm^−1^ are attributed to stretching vibrations of C-O and C-OH deformation vibrations. Several small peaks at 1292, 1340, and 1398 cm^−1^, and 1340, 1349, 1398, and 1419 cm^−1^, specific for WF/TEO, were attributed to the -C-H (CH_3_) symmetrical bending, while in the same region, fewer peaks were identified in WF/G4 and PLA spectra. At 1457 cm^−1^, the CH_3_ group scissoring vibrations and -CH2, -CH3 terminal group bending were present in all studied samples (Figure 4B).

The PCA analysis for the region 600 to 1500 cm^−1^ describing the fingerprinting shows that a single cluster is formed by the WF/TEO, WF/G1, WF/G2, and WF/G3, while the structure of WF/G3 shows a distinct intermediary structure between PLA and the rest of the films. PC1 explains 85% of the total variance and only 15% of the variance is explained by PC2 (Figure 4B1).

#### 3.1.3. Moisture Content (MC) and Solubility in Water

The moisture content of the films obtained ranged between 41.13 ± 3.28 in WF/G4 and 49.14 ± 1.85% in WF/TEO (Table 1) with significant differences (*p*-value = 0.009) only between WF/TEO and WF/G4.

Low values for water solubility indicate a resistance of the films against water molecule penetration, a feature necessary in commercial films [36]. The bio-based films exhibited values of solubility in water ranging between 63.67 ± 7.04 and 81.33 ± 6.45% (Table 1) and showed no significant differences (*p*-value = 0.033) among them, despite a noticeable decrease in water solubility correlated with an increase in film thickness from WF/G1 to WF/G4 and better mechanical strength. The lower moisture content of the composite with the increase in PLA mat thickness could be also positively correlated with the WVP decrease, WF/G4 showing the lowest moisture content and WVP from all tested formulae (Section 3.2.3). In various food packaging applications, a lower moisture content than in WF/G films would be necessary; thus, crosslinkers like transglutaminase or gluten fractions could be added to improve the composites’ structure and expand the applicability range.

The large-surface-area-to-mass ratio of the electrospun fiber mat [29] allows for the better accessibility of enzymes responsible for biodegradation in comparison with compact layers of PLA, which should accelerate biodegradability without impairing its flexibility.

Comparable solubility values were reported for WPC films with 40% glycerin [37] of 78.32 ± 0.13, but far lower solubility values (17.19 ± 0.32) than in our study, favored by the high glycerin content. A similar moisture content (40–42%) was reported [38] for composite films resulting from freeze powder mixture containing whey protein isolate and sunflower oil; but, in this case, different water solubilities from current ones were reported (47 to 55%).

### 3.2. Barrier Properties of Biocomposite Films

#### 3.2.1. Identification of Volatile Organic Compounds (VOCs) Released by the Films Covered with PLA Mat Using SMPE GC-MS Method

A total number of 14 VOCs originating from the TEO extract were tentatively identified in WF/G films (Table 2) using NIST library. A semi-quantitative method was applied to estimate the concentration of the major VOCs, using a procedure previously described [2,22]. The most abundant constituents of VOCs in the WF/G films is thymol, ranging from 50.65% in WF/G2 to 63.08% in WF/G4. Thymol concentration was reported as the highest in thyme essential oils by many studies [2,22,39]. Other major constituents tentatively identified in WF/G are o-cymene, 1-R α-pinene, and carvacrol, but also santolina triene. Researchers [40] found p-cymene (28.55%) and carvacrol (3.14%) alongside thymol (43.19%) in thyme essential oil. Minor compounds were also present in the WF/Gs and are indicated in Table 2.

It was obvious that composite WF/G films covered with PLA fiber mats of different thicknesses depending on electrospinning time and system configuration (1 needle and 2 needles) displayed different permeability against VOCs. As was expected, when increasing the time of electrospinning from 90 min in the case of WF/G1 to 210 min for WF/G3, the total VOC permeation through the film decreased by 23%, while WF/G4’ total VOC permeation was reduced by 17.71% in comparison with WF/G3. Thus, it can be concluded that a controlled permeation through the PLA fiber mat resulted when increasing the electrospinning time, but also when doubling the fiber application system using two face-to-face needles. Owing to the fact that, with the increase in the thickness of the PLA fiber mat, the diffusion of volatile compounds is reduced, additional protection against TEO loss in the environment and slower release rates of the active compounds are obtained. This could lead to a longer protection of the food covered by this bi-layer composite film in comparison with a mono-layer film with TEO.

It is interesting to notice from Table 2 that, for volatiles with KI from 854 to 875, characterized by high volatility, no significant differences (*p* > 0.05) were registered between WF/G1 and WF/G3, showing that only the mat created by the WF/G4 fiber is able to retain a big part of these highly volatile compounds and display significantly (*p* < 0.05) lower concentrations in the environment above.

**Figure 5:**
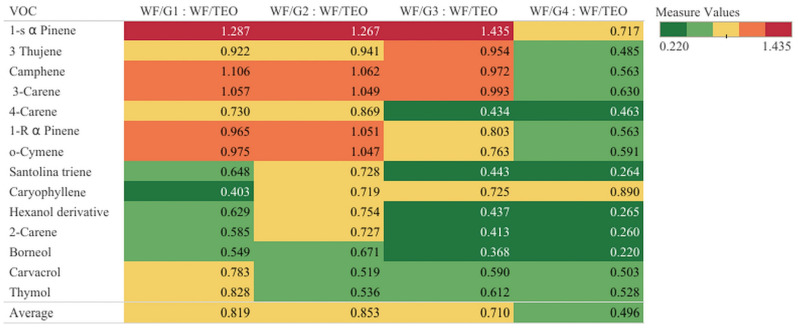
Heat map describing the retention capacity of WF/G films covered with PLA mat in comparison with the uncovered film (WF/TEO), where the red signifies no VOC retention and green very good barrier properties, while orange and yellow signify medium barrier properties of films.

To better understand the complex permeation process of VOCs through different PLA layers in comparison with the uncovered film WF/TEO, a heat map (Figure 5) was designed. The heat map uses color coding to illustrate the retention capacity of the composite films: green indicates reduced permeability to VOCs, yellow indicates moderate permeability, and red indicates high permeability and reduced capacity in retaining VOCs.

The VOCs in the first half of the heat map list, from 1-s αPinene to o-Cymene, are the ones with the highest volatility and are inclined to diffuse rapidly from the film structure. It should be noticed that low retention capacity was demonstrated by WF/G1, WF/G2 and WF/G3 that had a weak and similar retention capacity, indicating a high permeability of the PLA fiber mat to VOCs, as shown by the red and yellow colors. In contrast, WF/G4 composite exhibited a reduced permeability even for the very volatile compounds, with the exception of 1-s α Pinene, for which WF/G4 showed a medium permeability underlined by the yellow color.

It can be seen that low permeability was exhibited by WF/G4 for borneol, 2-carene, and santolina triene, which had a very low diffusion across the PLA mat, while for most of the VOCs, 40 to 60% of the VOCs permeated through the PLA mat. WF/G3 film also displayed a low permeability to borneol, carvacrol, and thymol; however, it presented a high permeability to thujene, pinene, camphene, and 3-carene.

In terms of the average capacity of WF/G films to control the permeability of the volatiles on the side covered by the PLA mat, WF/G1, WF/G2, and WF/G3 displayed a medium permeability score to volatiles, retaining only 20 to 30% of VOCs, while WF/G4 film displayed a 50% capacity to retain VOCs. Considering the volatile nature of TEO and its sensitivity to environmental factors, the capacity to control the permeability of the VOCs through the fiber mat represents an important asset that helps preserve the bioactivity of composite packaging materials, thereby improving food protection.

#### 3.2.2. Octanol Permeability by SMPE GC/MS

Permeability of 2-octanol across the PLA mat was assessed for all the samples in comparison with the control (WF/TEO) sample. The compound used as an internal standard, 2-octanol, is considered volatile mostly due to its molecular structure and physical properties. The molecular weight, the presence of the functional group (OH), and the intermolecular forces influence 2-octanol volatility.

**Figure 6:**
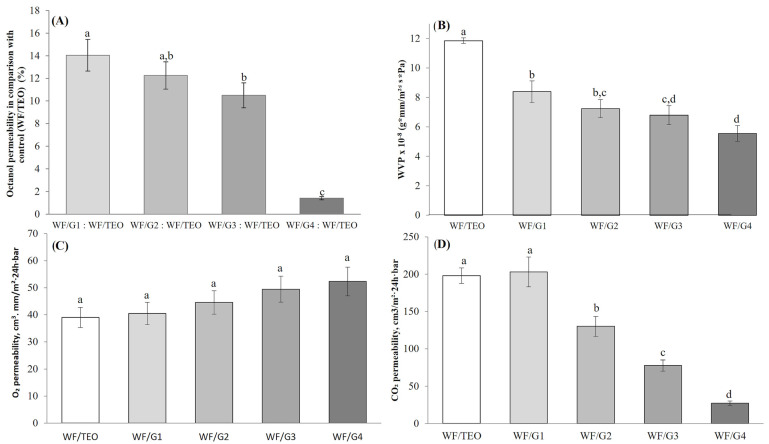
Permeability of the WF/G films: (**A**) 2-octanol determined by diffusion through the WF/G films positioned with PLA mat in comparison with control (WF/TEO) (*p*-value = 0.000); (**B**) water vapor (*p*-value = 0.000); (**C**) oxygen (*p*-value = 0.080); and (**D**) carbon dioxide (*p*-value = 0.000). Values obtained are the mean of three analyses ± SD. Significant differences among samples (*p* < 0.05) are indicated by different letters established by ANOVA and Tukey’s post hoc test.

The results (Figure 6A) indicate a lower permeability to 2-octanol shown by WF/G2, WF/G3, and WF/G4 composite films. A 1.15-, 1.33-, and 9.98-fold lower permeability, compared to WF/G1, which had the weakest barrier properties against octanol, was registered by WF/G2, WF/G3, and WF/G4. However, only 14.04 ± 1.40% of the permeability of the control was registered for the thinnest film with PLA mat (WF/G1), while, for the thickest film obtained with two face-to-face needles, permeability to 2-octanol dropped dramatically to 1.41 ± 0.14.

Designing new biopackaging-based materials implies creating a good barrier between the external environment and the food product, able to prevent migration and scalping, but it should also limit gas and vapor bidirectional exchanges. Food flavor alteration during storage could result from interactions with the packaging, leading to losses through permeation and/or migration. Permeation is a multistep process, involving collisions with the polymer, followed by sorption onto the polymer matrix, and then by migration through the polymer’ structure and, at the end, desorption [22].

The significant reduction in octanol permeability across all composite films (WF/G1, WF/G2, WF/G3, and WF/G4), mostly in WF/G4, compared to the control film is an indicator that composite materials with good barrier properties can be obtained using the PLA electrospun fiber mat.

#### 3.2.3. Water Vapor Permeability

WVP is one of the most important parameters in the selection of films as food packaging materials. Water can act as a solvent, thus causing the degradation of the texture and quality of the packaged food product, as well as chemical and enzymatic reactions in the structure of polymers used as packaging materials [41]. The WVP of the WF/G films covered with a PLA mat were determined and shown in Figure 6B. The results indicate a sharp decrease in WVP with the increase in the thickness of the PLA electrospun fiber mat (Figure 6B). The WVP registered a 18.57% decrease when the time for electrospinning increase from 90 min for WF/G1 to 210 min for WF/G3, and a 33.73% decrease when the fibers were produced with two face-to-face needles for 210 min for WF/G4. The results obtained in this study are in the same range as the ones reported in [42] for PLA/curcumin composites and by others [37]. Other studies outlined that the WVP value increases with the increase in the concentration of whey protein concentrate added to the composite film (5–10%) with pullulan and glycerol [43]. In this case, the WVP significantly decreased (*p* < 0.05) with the thickness increase in the less permeable material: PLA. These WVP values of the WF/G films are comparable, but lower than the ones for cellophane (9.9 × 10^−8^ g·mm/m^2^·s·Pa) and higher than the ones for LDPE (5.56 × 10^−10^ g·mm/m^2^·s·Pa) [44].

#### 3.2.4. Gas Permeability of EFs

In general, films based on whey protein are characterized by a fair oxygen barrier because of their highly polar nature and lower free space between polymer chains. The reinforcement with a PLA fiber mat showed a slight but non-significant (*p* > 0.05) increase in the barrier properties (Figure 6C). However, similar results were reported for the whey-based films reinforced with cellulose nanocrystals [45], where oxygen permeability did not change while WVP was reduced. The values of O_2_ permeability are slightly lower than the ones reported by researchers [46] for PLA thermoformed trays with cinnamaldehyde and carvacrol, and are comparable in the case of WF/TEO with the values reported by [37] for WPC films. Moreover, the oxygen permeability of WF/G materials is very close to that of PHA and EVOH [44]. The order of magnitude of EF/G films’ oxygen permeability is lower than of LDPE, HDPE, and PP values [44].

Permeability to CO_2_ markedly decreases with the increase in PLA layer thickness. Extending the electrospinning time from 90 min to 150 min decreased CO_2_ permeability 1.56-fold, and 2.62-fold from 90 min to 210 min. Using a dual-needle system further decreased CO_2_ permeability 7.42-fold in WF/G4 compared with WF/G1 (Figure 6D).

Oxygen and CO_2_ are non-polars, and there would be a dominance of diffusivity against the solubility through multilayer films and densely packed fibers resulting in a tortuous path for gas molecules. However, carbon dioxide has a significant quadrupole moment that shows a decreased permeability in comparison to oxygen, despite having similar kinetic diameters, because CO_2_ has a greater tendency towards hydrogen bond formation with OH groups available from terpenes and PLA; thus, it could become subsequently more soluble in the PLA mat [47]. In the case of water, the process of mass transfer is dominated by various interactions with the composites’ protein layer. In general, the homogeneous integration of PLA fibers contributed to better barrier properties of the composite films, reducing the water vapor permeability by up to ~34% and 10-fold for the permeability to CO_2_, while octanol permeability decreased 7.42-fold (Figure 6) in WF/G4 in comparison with WF/G1, which is attributed to the increased material density observed in SEM images (Figure 3).

### 3.3. Color and Opacity

The positive a* and b* values presented in Table 3 indicates yellow and red shades in the analyzed biocomposite materials, while the negative values for b* show the presence of blue tones only in the uncovered film with the PLA mat (WF/TEO). For all the CIELab parameters, WF/TEO (the control without a fiber mat) values are significantly different from the WF/Gs (with PLA fiber mat). When comparing WF/Gs with WF/TEO ∆E > 12 for all samples, one may notice significant color differences in comparison with the control [8]. However, non-significant color differences (*p* > 0.05) between the WF/G1, WF/G2, WF/G3, and the WF/G4 samples were registered. If the comparison is performed with WF/G1, then the ∆E for WF/G2, WF/G3, and WF/G4 shows significant differences (*p* < 0.05), while the ∆E for WF/G2 and WF/G3 is between 0.5 and 1.5, indicating a slight color difference; for WF/G4, the ∆E ranges between 1.5 and 3.0, indicating a significant color difference, which can be easily perceived by the naked eye.

The hue of the films did not differ significantly when increasing the thickness of the PLA fiber layer, although the yellow hue was decreased. Other authors [3] have studied the changes in color of the whey protein concentrate films with the addition of EOs (from 1 to 5%) and noticed that a characteristic yellowish tint of WPC-based films tends to increase with the addition of increasing concentrations of the EO blend due to its characteristic color. In this case, a higher but non-significant (*p* > 0.05) lightness was determined for the films with a thicker PLA mat (WF/G2, WF/G3, and WF/G4) in comparison with WF/G1. However, a significant (*p* < 0.05) but less intense chroma (Cab*) value was determined for the WF/G4 with the thickest PLA fiber mat.

The results indicate that the opacity of the biocomposite films increases with the thickness of the PLA layer applied by electrospinning (Table 3). The WF/G4 film is significantly different (*p* < 0.05) from WF/G1 and WF/G2. Compared to WF/G4, WF/G1, WF/G2, and WF/G3 films have a 1.38, 1.28, and 1.6 times lower opacity value, respectively. Films with higher opacity values can be an advantage for foods that are sensitive to external factors, for example, for those sensitive to the action of light able to generate photocatalytic oxidative reactions in food. Similar findings were reported [48] on PLA/TiO2 composite materials obtained by electrospinning, showing that the nanofibers applied to the films effectively act as a barrier to the penetration of UV light while increasing their opacity.

Even if food consumers would rather prefer a see-through material as packaging, in certain circumstances, when materials could be used as primary packaging (i.e., separators for slices) or in catering, it is not expected to negatively influence consumer opinion. Studies have shown that, when cream, brown, or green colors used in packaging were indicators of greater sustainability, it triggered likeability responses from consumers [49]. In addition, the non-transparent materials are able to better protect sensitive foods with high fat content against oxidation catalyzed by UV light. The light color of the composite WF/G materials next to its compatible composition with the food matrix makes them appropriate for different dairy applications like, for example, hard cheese.

### 3.4. Mechanical Properties

**Figure 7:**
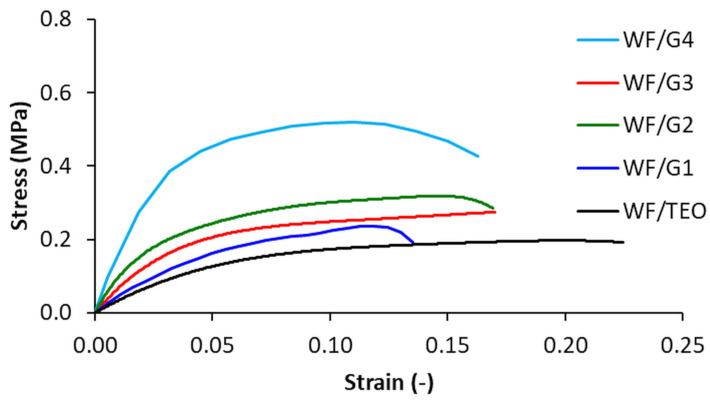
Representative stress–strain curves for biocomposite films.

Representative stress–strain curves for biocomposite films are shown in Figure 7. It should be noted that the elastic response and maximum stress have good repeatability, but the failure response shows high variations both in stress at break and elongation.

The stress–strain curves in Figure 7 indicate that the mechanical properties are dependent on the electrospinning time and the number of pumps used for the electrospinning of PLA fibers. The WF/G4 films, for which a dual-pump system was used, show a significantly improved tensile strength and stiffness in comparison with the WF/G3 films, for which only one pump was used. The PLA fiber mat reduces the ductility of the WF/G films as compared to the WF/TEO films, which exhibited the highest elongation at break of about 22.5%. It should be noted that the elastic response and maximum stress have good repeatability, but the failure response shows high variations both in stress at break and elongation.

In terms of mechanical properties, the main function of the PLA fiber mat is to improve the Young’s modulus and tensile strength of the films. Table 4 indicates that the Young’s modulus of the WF/G films increases with extended electrospinning time, which directly correlates to the increased thickness of the PLA nanofibrous layer.

Experimental results indicate up to a 159% increase in stiffness as the electrospinning time increased from 0 to 210 min. Adding a second pump resulted in a considerable increase in film stiffness. The WF/G4 film had the highest Young’s modulus (5.16 times higher than WF/TEO and about 2.0 times higher than WF/G3), and this is explained by the fact that, in the case of the WF/G4 film, the thickness of the PLA fiber mat is 3.44 times higher than of the WF/G3 film, which has a significant contribution to the overall stiffness.

It should be noted that the values of Young’s modulus reported in this study are close to the values reported for the PLA mat with 18.0% oligomeric lactic acid used as plasticizer and 1.8% content MgO nanoparticles [50]. Similarly, ref. [33] reported comparable Young’s modulus values for zein electrospun fibers loaded with 20% concentration of savory essential oil with WF/G4, but about four-times-higher tensile strength. Researchers [51] reported comparable tensile strength with the current study, but higher values for Young’s modulus for zein-basil seed gum electrospun nanofibers activated with thyme essential oil.

For the films fabricated with one syringe and one pump, the tensile strength increased by about 81.5% as electrospinning time rose from 0 to 210 min, as shown in Table 4. Again, the WF/G4 film had the highest tensile strength (about 2.70 times higher than WF/TEO, and 1.49 times higher than WF/G3) as the load bearing capacity was increased by the PLA nanofibrous layer.

Generally, whey protein films have a high water hydration capacity that weakens their mechanical properties. Considering the inherent brittleness of WF/Gs, crosslinkers could also be included in order to enhance mechanical properties [52] in some specific applications where resistance should be the prevailing feature of the composite materials.

In conclusion, the PLA mat applied on one side of the composite WF/G displayed an increased thickness with the increase in the electrospinning time and the homogeneous fibers in a +/− 75° angle distribution from the collector’s generator, which correlates with an improvement in the tensile strength of the films (Table 4).

### 3.5. Characterization of Whey Films Functionalized with TEO (WF/TEO)

#### 3.5.1. Antimicrobial Activity of Films

The antimicrobial activity was estimated on the active side of the WF/G films with no significant differences (*p* > 0.05) noticed between the WF/G films and WF/TEO for the disc diffusion method. It was observed that WF/TEO inhibited the development of microorganisms, showing different inhibition diameters depending on the test microorganism (Table 5).

WF/TEO exerted an inhibitory effect against all tested microorganisms, with inhibition diameters ranging from 1.00 ± 0.15 mm (*L. monocytogenes*) to 45.47 ± 0.25 mm (*G. candidum*), with significant differences (*p* < 0.05) noticed among the studied microorganisms. The highest inhibitory effect was against *G. candidum*, followed by *B. cereus*, and approximately 50% of the higher antimicrobial activity was registered for *R. glutinis*. The antimicrobial effect of WF/TEO and WF/Gs was classified as previously indicated by [2] as strong due to zones of inhibition higher than 20 mm for *R. glutinis*, *B. cereus*, and *G. candidum*, and as having no inhibitory effect for zones of inhibition less than 12 mm, as observed for *L. monocytogenes* Scott A. The antimicrobial activity of thymol and carvacrol, the main components of thyme present in the WF/G composites, is related to their chemical structures, which contain delocalized electrons and hydroxyl groups that can act as proton exchangers and modify the equilibrium of inorganic ions in the cytoplasm of bacteria [33].

This was also demonstrated [33] in zein electrospun fibers loaded with savory essential oil where, beside carvacrol, γ-terpinene and p-cymene were responsible for the antimicrobial activity. In our case, as a result of the PLA mat applied, most probably all the WF/G films had mixtures of active volatiles (Figure 5) containing high concentrations of carvacrol and thymol (WF/G2, WF/G3, and WF/G4) and caryophyllene, 3-carene, and borneol (WF/G1), that, in total, can exert a comparable (*p* > 0.05) effect on certain tested microorganisms, presented in Table 5. This would recommend any of the tested formulae to be applied as active layers for compatible food matrices.

#### 3.5.2. Antiradical Activity of Films

**Figure 8:**
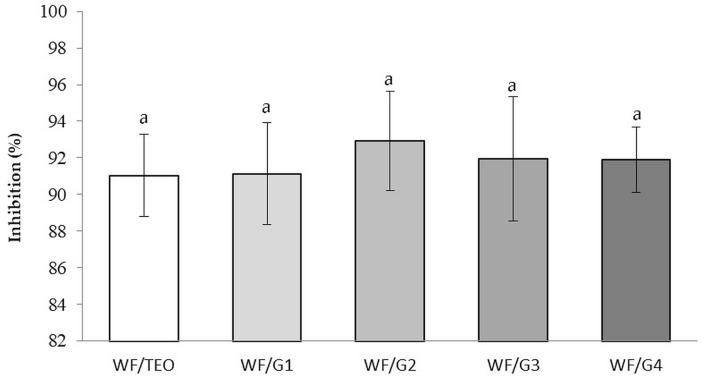
Antiradical activity of film without TEO (control WF) and with TEO (WF/TEO). Values obtained are the mean of three analyzes ± SD; different letters obtained indicate significant differences (*p* < 0.05) between samples as determined by ANOVA and Tukey’s post hoc test; *p*-value = 0.000.

Active antioxidant packaging is used to extend the shelf life of foods and minimize exposure to oxidative stress, especially of foods rich in oxygen-sensitive fats [53]. Non-significant differences (*p* > 0.05) were registered between WF/G1, WF/G2, WF/G3, and WF/TEO, demonstrating that covering films with a PLA fiber mat did not change the antiradical activity. WF/TEO presented a free radical inhibition value of 89.03 ± 8.9% (Figure 8). This is also related to the comparable capacity of WF/G films to retain active molecules such as carvacrol, thymol, 2-carene, and borneol, as shown in Figure 5. The main volatiles of TEO, thymol and carvacrol, acted as hydrogen donors that stabilize free radicals by forming stable phenoxy radicals, which explains the high antiradical activity observed.

Researchers [54] also demonstrated that fibers loaded with carvacrol had antioxidant activity against DPPH, ranging from 20.0 to 52.6%.

In another study, ref. [53] obtained an antiradical activity of 79.4%, 83.3%, and 84.3% for films containing TEO, oregano, and rosemary, respectively. These results have the same order of magnitude as those of the present study, showing a strong antiradical effect produced by the terpenes present in the films.

The antimicrobial and antioxidant activities displayed by the WF/G films do not vary significantly in spite of the differences in thickness of the PLA mat, most probably due to controlled permeation that slowed down the diffusion of the most active volatile compounds like carvacrol and thymol through the composite.

The antimicrobial and antioxidant active properties of the composite protein film reinforced with a PLA fiber mat and TEO qualify the material for various applications in tissue engineering, as suggested in a recent review [55]. Applications of electrospinning technology in conjunction with plant essential oils also present noteworthy advantages for prolonging the shelf life of perishable fruits and vegetables [56]. Additionally, food matrices compatible with thyme flavor, such as hard cheese, could also demonstrate biocompatibility, providing stability and prospects for developing new types of dairies.

## 4. Conclusions

In this research, composite films of whey proteins with TEO and PLA electrospun fiber mat were obtained and characterized. Electrospinning was used to cover the film with a PLA fiber mat, varying the electrospinning time up to 210 min and the number of needles/pumps. The vibrations of the functional groups of the WF/TEO were shielded as the thickness of the PLA layer increased. Moreover, the thickest film obtained with two face-to-face needles after 210 min retained almost 50% of the total VOCs including the most volatile compounds (thujene, camphene, o-cymene) and displayed the highest barrier properties against octanol; WVP and CO_2_ and showed the highest opacity of all tested formulae. To evaluate the properties of VOC diffusion through the films, an original method was developed for SPME GC/Ms. The biocomposite films exhibit constantly increasing stiffness and tensile strength as a result of the increasing PLA nanofibrous layer thickness. Experimental results for the film with the thickest PLA fiber mat show up to a 159% increase in stiffness and about an 80% increase in tensile strength in comparison with the uncovered film. In addition, a considerable increase in the mechanical properties of the films is observed by adding a second pump, providing films with the highest Young’s modulus (5.16 times higher than WF/TEO) and a 2.71-times-higher tensile strength compared to WF/TEO as a result of the increasing PLA nanofibrous layer thickness.

The functional properties of all WF/Gs given by the volatile terpenes (thymol, carvacrol, camphene, α-pinene, etc.) embedded in TEO films demonstrated good antiradical activity and strong antimicrobial activity against *B. cereus* and *G. candidum*. Moderate inhibitory activity was observed against *R. glutinis*, while weak inhibitory activity was recorded against *L. monocytogenes Scott A.* The weak antilisterial activity of WF/Gs could be improved by adding higher TEO concentration, although this should also be correlated with a balanced flavor intensity. Based on the results regarding VOC controlled permeation through the PLA fiber mat, it was proven that bi-layered biocomposite films have good potential for being used as active packaging materials. Optimized formulae of composite bio-based films could contribute to the development of sustainable packaging materials for the food industry and offer alternatives to the current conventional plastics. However, future studies should explore the biocompatibility with various food matrices to expand their potential applicability, with one direction being hard cheese.

Furthermore, a comprehensive perspective will be given by expanding the current knowledge with a larger screening of antimicrobial activity against critical pathogens, food packaging stability tests, and biodegradability tests of the composite materials.

Industrial applications are, for the time being, limited by the challenges in upscaling the electrospinning; however, we expect these limitations are soon to be tackled.

## Figures and Tables

**Table 1:** Moisture content and water solubility.

Films	MC (%)	S (%)
WF/TEO	49.14 ± 1.85 ^a,^*	81.33 ± 6.45 ^a,^*
WF/G1	48.59 ± 3.15 ^a^	80.66 ± 7.23 ^a^
WF/G2	40.36 ± 2.62 ^a,b^	78.15 ± 8.22 ^a^
WF/G3	41.34 ± 4.06 ^a,b^	66.28 ± 6.92 ^a^
WF/G4	41.13 ± 3.27 ^b^	63.67 ± 7.04 ^a^

* on columns, different letters confirm significant differences (*p* < 0.05) by post hoc Tuckey test.

**Table 2:** The relative concentration of VOCs (μg octanol/ cm^3^) released by the WF/G films covered with the PLA fiber mat determined by SPME GC/MS.

Compound	KI	WF/G1	WF/G2	WF/G3	WF/G4	*p*-Value
1-s α Pinene	854	55.91 ± 6.28 ^a,^*	55.04 ± 5.55 ^a^	62.35 ± 6.89 ^a^	31.15 ± 4.45 ^b^	0.012
3 Thujene	859	43.80 ± 4.66 ^a^	44.69 ± 4.88 ^a^	45.31 ± 5.24 ^a^	23.01 ± 2.41 ^b^	0.007
Camphene	887	51.28 ± 4.48 ^a^	49.20 ± 5.29 ^a^	45.03 ± 3.17 ^a^	26.09 ± 3.35 ^b^	0.003
3-Carene	862	112.51 ± 12.42 ^a^	111.62 ± 11.96 ^a^	105.72 ± 11.34 ^a^	67.06 ± 7.59 ^b^	0.017
4-Carene	875	75.64 ± 6.65 ^a^	90.01 ± 10.11 ^a^	44.96 ± 5.8 ^b^	47.93 ± 6.22 ^b^	0.004
1-R α Pinene	934	1023.34 ± 100.12 ^a^	1114.35 ± 105.68 ^a,b^	851.98 ± 67.55 ^b^	596.80 ± 55.36 ^c^	0.001
o-Cymene	954	1723.55 ± 112.38 ^a^	1850.31 ± 159.32 ^a^	1349.68 ± 122.48 ^b^	1044.19 ± 128.15 ^b^	0.001
Santolina triene	1166	186.09 ± 20.01 ^a^	208.70 ± 17.23 ^a^	127.05 ± 13.14 ^b^	75.85 ± 6.49 ^c^	0.000
Caryophyllene	1189	114.07 ± 10.59 ^b^	203.39 ± 23.87 ^a^	204.92 ± 19.98 ^a^	251.78 ± 23.74 ^a^	0.001
Hexanol derivative	1191	70.03 ± 7.88 ^a^	83.88 ± 7.36 ^a^	48.70 ± 3.99 ^b^	29.53 ± 2.18 ^c^	0.000
2-Carene	1401	22.91 ± 1.45 ^b^	28.47 ± 2.04 ^a^	16.18 ± 1.93 ^c^	10.16 ± 1.27 ^d^	0.000
Borneol	1402	132.97 ± 14.23 ^b^	162.55 ± 1.45 ^a^	89.13 ± 6.27 ^c^	53.34 ± 4.33 ^d^	0.000
Thymol	1436	6823.34 ± 592.48 ^a^	4413.67 ± 359.16 ^b^	5046.88 ± 314.62 ^b^	4347.75 ± 428.97 ^b^	0.001
Carvacrol	1449	447.26 ± 38.25 ^a^	296.57 ± 28.44 ^b^	337.22 ± 25.81 ^b^	287.50 ± 20.09 ^b^	0.000

* On rows, different letters show significant differences (*p* < 0.05) by post hoc Tuckey test; KI—Kovats Index; *p*-value determined by ANOVA.

**Table 3:** Color parameters of biocomposite films with PLA mat oriented upwards and WF film with TEO oriented downwards.

	Parameters	L^*^	a^*^	b^*^	H_ab_ (°)	C_ab_	∆E _WF/TEO_	∆E _WF/G1_	O (%)
Sample									
WF/TEO		45.17 ± 1.69 ^b,^*	0.12 ± 0.02 ^b^	−0.20 ± 0.03 ^d^	120.96 ± 10.5 ^a^	0.23 ± 0.01 ^d^	-	-	ND
WF/G1		64.46 ± 4.58 ^a^	1.51 ± 0.18 ^a^	6.09 ± 0.02 ^a^	75.81 ± 3.12 ^b^	6.29 ± 0.62 ^a^	19.99 ± 1.03 ^a^	-	58.04 ± 5.50 ^b^
WF/G2		63.33 ± 0.04 ^a^	1.85 ± 0.16 ^a^	5.45 ± 0.47 ^a,b^	70.96 ± 4.13 ^b^	5.67 ± 0.57 ^a,b^	19.05 ± 0.98 ^a^	1.12 ± 0.0 ^c^	62.72 ± 6.10 ^b^
WF/G3		64.35 ± 1.13 ^a^	1.55 ± 0.01 ^a^	4.71 ± 0.01 ^b,c^	71.75 ± 4.19 ^b^	4.95 ± 0.49 ^b,c^	19.98 ± 0.87 ^a^	1.42 ± 0.02 ^b^	69.17 ± 7.20 ^a,b^
WF/G4		64.20 ± 0.81 ^a^	1.71 ± 0.02 ^a^	4.06 ± 0.06 ^c^	66.85 ± 5.47 ^b^	4.35 ± 0.42 c	19.56 ± 0.79 ^a^	2.10 ± 0.04 ^a^	80.44 ± 8.10 ^a^
*p*-value		0.000	0.000	0.000	0.000	0.000	0.580	0.000	0.018

* The values obtained are the mean of three replicates ± SD; different letters obtained indicate significant differences (*p* < 0.05) between samples, as determined by ANOVA and Tukey’s post hoc test; *p*-value determined by ANOVA.

**Table 4:** Mechanical parameters of biocomposite films.

Films	Time (min)	Young Modulus (MPa)	Tensile Strength (MPa)	Film Thickness (mm)	PLA Layer Thickness (µm)
WF/TEO	0	2.39 ± 0.04 ^d,^*	0.206 ± 0.004 ^c^	0.369 ± 0.010 ^c^	-
WF/G1	90	4.04 ± 1.06 ^c,d^	0.214 ± 0.038 ^c^	0.404 ± 0.010 ^b,c^	9.623 ± 2.857 ^c^
WF/G2	150	5.16 ± 1.02 ^b,c^	0.328 ± 0.004 ^b^	0.404 ± 0.011 ^b,c^	11.122 ± 0.527 ^c^
WF/G3	210	6.20 ± 0.46 ^b^	0.374 ± 0.019 ^b^	0.419 ± 0.015 ^b^	20.325 ± 1.147 ^b^
WF/G4	210	12.34 ± 0.02 ^a^	0.557 ± 0.041 ^a^	0.468 ± 0.031 ^a^	69.905 ± 2.347 ^a^
*p*-value	-	0.000	0.000	0.001	0.000

* The values are the mean of three replicates ± SD; different letters indicate significant differences (*p* < 0.05) between the WF/G4 samples and WF/G1, WF/G2, and WF/G3, as determined by ANOVA and Tukey’s post hoc test; *p*-value determined by ANOVA.

**Table 5:** Inhibition zones of films with TEO.

Test Microorganism	WF/TEO	WF/G1	WG/G2	WF/G3	WF/G4
	(mm)				
*B. cereus*	38.99 ± 0.36 ^a,^*	37.64 ± 0.77 ^a^	37.39 ± 0.72 ^a^	37.53 ± 0.48 ^a^	37.26 ± 1.07 ^a^
*G. candidum*	45.47 ± 0.25 ^b^	45.05 ± 0.72 ^b^	44.87 ± 0.66 ^b^	44.31 ± 0.22 ^b^	44.76 ± 1.09 ^b^
*R. glutinis*	20.71 ± 0.53 ^c^	21.15 ± 0.35 ^c^	20.91 ± 0.39 ^c^	20.12 ± 0.08 ^c^	20.51 ± 0.54 ^c^
*L. monocytogenes*	1.00 ± 0.15 ^d^	0.99 ± 0.02 ^d^	0.93 ± 0.09 ^d^	0.88 ± 0.09 ^d^	0.88 ± 0.06 ^d^
*p*-value	0.000	0.000	0.000	0.000	0.000

* The values obtained are the mean of the three analyses ± SD; significant differences (*p* < 0.05) on columns are indicated by different letters as determined by ANOVA and Tukey’s post hoc test; *p*-value determined by ANOVA.

## Data Availability

Dorofte, A., Bleoanca, I., Bucur, F.I., Mustatea, G., Borda, D., Stan, F., Fetecau, C., 2024. Whey-based films with PLA fiber mat [dataset]. Mendeley data, v1. https://doi.org/10.17632/s3wc7nc5s3.1, (accessed on 12 November 2024).
